# Active AU Based Patch Weighting for Facial Expression Recognition

**DOI:** 10.3390/s17020275

**Published:** 2017-01-30

**Authors:** Weicheng Xie, Linlin Shen, Meng Yang, Zhihui Lai

**Affiliations:** Computer Vision Institute, School of Computer Science & Software Engineering, Shenzhen University, Shenzhen, Guangdong 518060, China; wcxie@szu.edu.cn (W.X.); yang.meng@szu.edu.cn (M.Y.); laizhihui@szu.edu.cn (Z.L.)

**Keywords:** expression recognition, expression triplet, feature optimization, AU weighting, active AU detection

## Abstract

Facial expression has many applications in human-computer interaction. Although feature extraction and selection have been well studied, the specificity of each expression variation is not fully explored in state-of-the-art works. In this work, the problem of multiclass expression recognition is converted into triplet-wise expression recognition. For each expression triplet, a new feature optimization model based on action unit (AU) weighting and patch weight optimization is proposed to represent the specificity of the expression triplet. The sparse representation-based approach is then proposed to detect the active AUs of the testing sample for better generalization. The algorithm achieved competitive accuracies of 89.67% and 94.09% for the Jaffe and Cohn–Kanade (CK+) databases, respectively. Better cross-database performance has also been observed.

## 1. Introduction

With the help of facial expression recognition, human-computer interaction can automatically obtain the information of the human face and infer the psychological status of the user, which can be applied to driver monitoring, face paralysis expression recognition, intelligent access control, and so on.

Recognition of six basic expressions, like happy (Ha), angry (An), surprise (Su), fear (Fe), disgust (Di), sad (Sa) and neutral (Ne) expression, can be categorized into 3D-based and 2D-based approaches. The 3D-based expression recognition is a current research hot topic [1], which often employs the geometry features, like differential curvature [2,3], based on an aligned face mesh [4]. The 2D-based approaches are currently prevailing due to the easy accessibility of the training samples. The facial action coding system (FACS) [5] is one of the important 2D approaches, i.e., the expressions are described and tracked according to some basic action units (AUs). The FACS was defined by Ekman [6] to reflect the deformation status of the corresponding facial part, which was developed based on a set of discrete emotions and initially applied to measure some specific facial muscle movements named AUs. While AUs were often used as an intermediate step for recognizing the basic expressions, image-based feature representation is often considered for recognizing the expression directly. Dynamic recognition based on expression images is important in face animation and multimedia analysis [7,8,9,10]. As less information about the considered expression is available, static image-based recognition is more challenging.

Deep learning with a convolutional neural network (CNN), such as multiscale feature-based CNN [11], hierarchical committee-based CNN [12] and architecture-improved CNN [13], has also been applied for static expression recognition. Pramerdorfer and Kampel [14] gave a detailed survey about these algorithms. Although it is experimentally verified in [15] that visually similar features to the facial AUs are obtained by the CNN-based algorithms, the weighting and optimization of these AU alike features in these algorithms are not fully studied. Meanwhile, CNN-based algorithms require a large number of training parameters and high computational complexity [16,17]. Thus, our work focuses on exploring the feature optimization and active AU detection, which can be further integrated into the CNN to further improve the encoded features.

For static image encoding, many features have been proposed and evaluated. Examples are the Gabor surface feature [18], Haar-like features [19], histograms of oriented gradients (HOG), local binary patterns (LBP) [20,21], the radial encoding feature [22] and the key point movement feature [23]. The combination of texture and geometric features was introduced [24] to solve the problem of minor expression deformation when the wrinkle feature is unavailable [25]. In this work, the Gabor surface feature (GSF) proposed in [18] is employed for the feature representation. The Gabor magnitude surface can reflect the differential geometry information even when the considered face is slightly deformed. Thus, it can discriminate different wrinkle textures on the expression face.

Based on the devised features, feature selection was often conducted for not only boosting the efficiency [26], but also improving the recognition accuracy and the generalization ability [27,28]. Feature selection can be conducted on the whole image, such as independent component analysis (ICA) [29], linear discriminant analysis (LDA) [30], rotational invariant dimensionality reduction [31], maximum margin projection [32] and supervised locally linear embedding [33]. For patch and landmark point-based feature representation, salient expression regions were manually located [34]. Automatic feature selection was usually realized with AdaBoost [19,20,26,35]. For better generalization ability, some feature selection algorithms attempted to obtain a relatively sparse number of features with the incorporation of optimization algorithms. Jia et al. [36] weighted the LBP feature with sparse representation, Zafeiriou and Pitas [37] proposed the sparse feature graph for the recognition. Feature selection has also been achieved by feature reduction or transformation, such as margin maximization [38], normalized cut [28], multitask joint sparse presentation [39] and locality preserving projection (LPP) [40]. A unified classification system [41] integrating feature selection and reduction was proposed based on the boosted deep belief network (BDBN).

These algorithms devised and selected the same features for all of the categories of expressions (such as six basic expressions), which may leave out the feature specialty and multi-scale property. Thus, pairwise expression features were proposed to improve the recognition performance. Kyperountas et al. [42] employed a pairwise expression recognition with a class separability measure by pairwise inter-class difference maximization and intra-class difference minimization. Happy and Routray [43] extracted pairwise appearance features with LDA for the recognition. Based on different pairwise expression features, the feature selections were also different. Liu et al. [44] proposed the feature disentangling machine (FDM) to learn different pairwise features. Besides the common features for all of the expressions, the specific features for each expression pair were also selected with multi-task learning in [45]. When most of these algorithms extract expression features from patches, they may not fully consider the causal relation between the patches since facial expressions are often demonstrated in the scale of facial parts involving multiple patches.

The AU-based features integrate the causal relation of patch features naturally since they reflect the deformation status of the facial parts. Tian et al. [5] used the classified AUs for the facial expression recognition, where the AUs were encoded by the geometric size and deformation. Tong et al. [46] constructed the Bayesian network of the causal relation of facial AU features with the corresponding conditional probability table. The expression appearance variance is represented with the assembly of AUs by the deep network [47]. Zhao et al. [48] proposed the component feature based on face block and weight assignment for expression recognition from near-infrared videos. The AU deformation intensities were estimated with regressors and used to train different classifiers for expression recognition [34]. Li et al. [49] introduced a unified probabilistic framework to simultaneously represent the facial AU evolvement, interactions and observations with different levels of feature representations and the classification system.

However, current AU-based algorithms modeled the AU relations without considering the weights of patches contained in each AU. The AU feature is suitable for encoding the macro-scale information of each expression, since it integrates the large-scale information of face parts. However, they are not good for encoding the micro-scale feature, since the combination space of AUs is limited when they are not carefully organized. Moreover, most of these algorithms learned the features from the training expression samples. However, the active feature implied in each testing expression sample is not fully exploited. The model learned from training samples might produce the wrong classification when the testing sample is significantly different. However, only a few works detect and use the active features of the testing sample for recognition.

Although many research works have been conducted in face expression recognition, the following problems are still to be addressed. First, current algorithms recognize the seven expressions entirely with a uniform feature weight, which may leave out the feature specialty and multi-scale property. In this work, the seven expressions’ recognition is divided into multiple sub-problems with appropriate subsets, i.e., the expression triplet. Moreover, the weight vector w.r.t. each expression triplet is fine tuned individually to fully consider its specificity. Second, the patch-based and AU-based features are often encoded separately without considering the influence of their deficiencies. In this work, the weights of patches contained in each AU are finely optimized to represent the characteristics of different expressions. In this way, the advantages of large-scale (AU-based) and small-scale (patch-wise) features are both explored. Third, few of the current works make use of the specificity of each testing sample before recognition; the wrong classification could occur when the testing sample is significantly different. Thus, this work exploits the active features of each testing expression.

The main novelties of this work are mainly on three aspects. First, a two-stage expression recognition using the idea of expression triplet weighting is introduced for the representation of diversity among different expressions. Second, a new offline weight optimization for the patches contained in each AU is proposed to increase the discrimination abilities of both the patch and AU features. Third, online detection of active AUs for each testing sample is proposed to fully exploit its specificity for feature encoding.

This paper is structured as follows. Section 2 gives a description of the proposed algorithm step by step. The experimental results of the proposed algorithm on public databases are presented in Section 3. Finally, discussions and some conclusions are addressed in Section 4.

## 2. The Proposed Algorithm

### 2.1. Framework of the Algorithm

**Figure 1:**
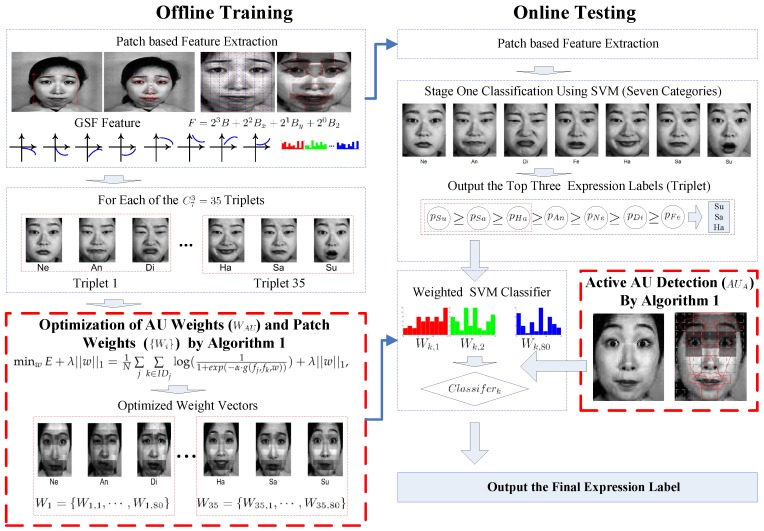
The framework of the proposed algorithm.

The sketch of expression recognition is illustrated in Figure 1. In offline training, faces were divided into a number of non-overlapped patches, regions and facial parts, like eyes, nose and mouth, where Gabor surface features were extracted. AUs for each face part are defined, as well. For each expression triplet combination, the weights of AUs and patches were optimized. In testing, the standard seven class-based expression recognition was conducted at the first stage, and the top three expression candidates were proposed. Based on the suggested expression triplet, the active AUs of the testing sample were detected and weighted using the learned weight vector. Weighted SVM was finally applied to give the expression label. The entire algorithm is then elaborated in the following sections.

### 2.2. Region Definition and Feature Extraction

#### 2.2.1. Definition of Patch, Region, Part and AU

The face image was first aligned with reference to the feature points located using the approach presented in [50,51] and then resized to 84×68. Only the central region with the size of 80×64 is cropped out for the following processing. Illumination was normalized by the method proposed in [52].

**Figure 2:**
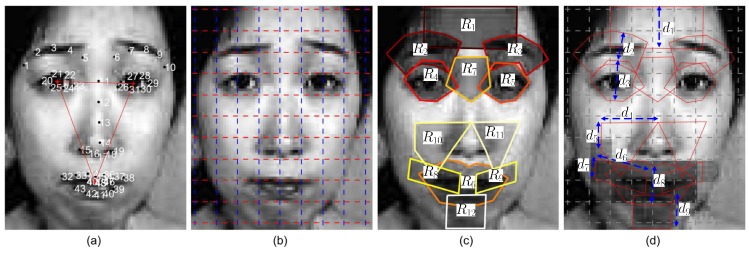
The patches and relatively fixed shape (RFS) regions. (**a**) The landmarks used for alignment; (**b**) the patches; (**c**) the RFS regions for defining face parts; (**d**) the sizes $d_{1}-d_{9}$ of the RFS regions. The darker region on the mouth part denotes the patches having a nonempty intersection with $R_{6},\;R_{8},\;R_{9}$ in (**c**), which presents an example of the relation between patch, region and part.

**Figure 3:**
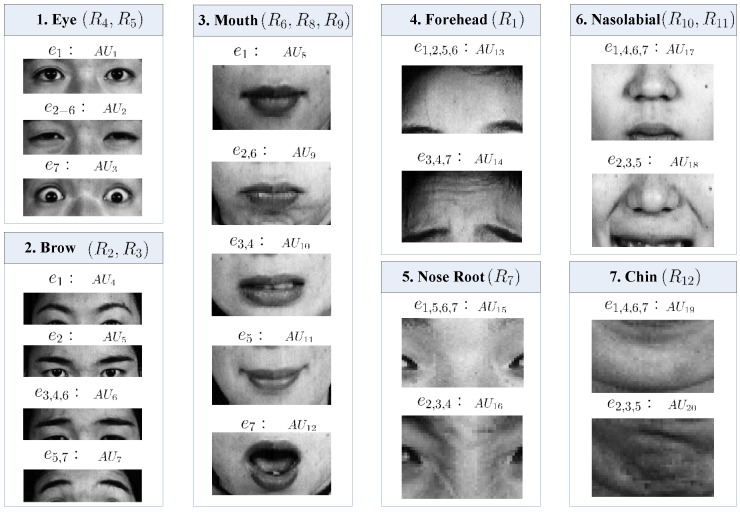
The AUs of seven facial parts with the corresponding RFSs and expression labels. The first part (eye, $PT_{1}$) consists of two regions $R_{4},\;R_{5}$, which can be classified as $AU_{1}-AU_{3}$ according to the eye status. The listed $e_{1}-e_{7}$ are the expressions related with each defined AU, i.e., neutral, angry, disgust, fear, happy, sad and surprise.

To extract features representing local face variations caused by expression, the face image was divided into 10×8 patches. Based on the 80 patches, 12 regions with relatively fixed shapes (RFSs) were defined to represent the expression-sensitive face parts (PT). Figure 2c shows the 12 regions, and Table 1 lists the involved landmarks for each region. When patches are used to represent local texture, regions encode the important variations correlated with expression changes. Figure 3 presents the involved regions used to define each of the seven face parts, i.e., eyes, brows, mouth, forehead, nose root, nasolabial regions and chin ($PT_{1}-PT_{7}$).

For encoding the face part variations related with expression [53], AUs have been widely used in the literature. We defined 20 AUs to encode the seven expressions named as $e_{1}-e_{7}$, i.e., neutral, angry, disgust, fear, happy, sad and surprise. Different from the AU labeling in [53], the part regions of each AU are manually labeled for training samples in this work. See Figure 3 for the definition of the 20 AUs and their relationship with the 12 regions and the seven parts. For example, $AU_{3}$ encodes wide open eyes, which is usually correlated with $e_{7}$, i.e., surprise. As both $AU_{1}$ and $AU_{2}$ encode the status of the two eyes, they mainly consist of regions around eyes, i.e., $R_{4}$ and $R_{5}$. Figure 2d labels the involved patches of an example mouth part ($AU_{8}-AU_{12}$) with dark grey, i.e., each AU of a part may involve multiple patches.

#### 2.2.2. Feature Extraction

For each patch or RFS region, the Gabor filter [54] is employed for feature extraction, which is formulated as:

(1) $$\left\{\begin{array}{c} g\left(x_{1},x_{2}\right)=\frac{k_{s}^{2}}{\sigma^{2}}exp\left[-\frac{k_{s}^{2}}{2\sigma^{2}}\left({x_{1}^{\prime }}^{2}+{x_{2}^{\prime }}^{2}\right)\right]exp\left(k_{s}x_{1}^{\prime }i\right)\\ x_{1}^{\prime }=\cos\alpha_{a}x_{1}+\sin\alpha_{a}x_{2},x_{2}^{\prime }=-\sin\alpha_{a}x_{1}+\cos\alpha_{a}x_{2},\\ k_{s}=\frac{\pi }{{\sqrt{2}}^{2+s}},s=1,\cdots ,n_{s};\alpha_{a}=\frac{a\pi }{8},a=1,\cdots ,n_{a}. \end{array}\right.$$

where σ=1.8π, $n_{s}=5,n_{a}=8$, $\left(k_{s},\alpha_{a}\right)$ define the amplitude and orientation of the central frequency.

To encode the texture magnitude map, the Gabor surface feature (GSF) [18] is employed, since it can depict the curvature information of the wrinkles and the direction of the expression texture. To extract GSF, the $n_{s}\times n_{a}$ Gabor magnitude images are first extracted, which are then encoded by the local binary pattern (LBP) to reduce the feature sensitivity to misalignment. More precisely, feature GSF for the *k*-th patch $p_{k}$ is formulated as follows:

(2) $$pf_{p_{k}}=2^{3}B+2^{2}B_{x}+2^{1}B_{y}+2^{0}B_{2},$$

where $B,\;B_{x},\;B_{y},\;B_{2}$ are the binarizations of the Gabor magnitude image *I*, its first-order and second-order gradient pictures $I_{x}$, $I_{y}$, $I_{xx}+I_{yy}$ corresponding to the patch $p_{k}$. As an example, for each pixel (i,j) of $p_{k}$, its binary value is defined as:

(3) $$B_{i,j}=\left\{\begin{array}{cc} 1 & \mathrm{if}\;I_{i,j}\geq ThresMed_{i,j}\\ 0 & \mathrm{otherwise} \end{array}\right.$$

where the threshold $ThresMed_{i,j}$ is the median of the pixel values of patch $p_{k}$. Thus, $pf_{p_{k}}$ is the output feature map with the value ranging from zero to 15, which is further transformed to the histogram for feature representation. For each face patch or region, the corresponding feature GSF is then vectorized as a $16\times n_{s}\times n_{a}$ dimension vector, where $n_{s},\;n_{a}$ are defined in Equation (1). Finally, the feature of the *i*-th expression sample is represented as:

(4) $$f_{i}=\left(pf_{p_{1}},\cdots ,pf_{p_{n}}\right),$$

where *n* is the number of patches or regions. For convenience of the following illustration, the feature of the *i*-th expression sample can be also grouped as $\left(f_{i}^{\left(1\right)},\cdots ,f_{i}^{\left(7\right)}\right)$ according to the seven face parts presented in Figure 3.

### 2.3. Feature Optimization

Based on the feature representation, the weights of the AU and patches for each expression triplet are optimized. First, the original AUs are weighted with the conditional transition probability matrix, based on which, weight optimization is performed to weigh the patches involved in each AU in the second step. These two steps are conducted on the training samples and are offline. The third step is to select the active AUs for each testing sample. The entire procedure of the feature optimization is presented in Algorithm 1.

**Algorithm 1** AU weighting, patch weight optimization and active AU detection.1:**Offline Training:**2:AU weighting ($W_{AU}$) using the conditional probability matrix presented in Section 2.3.1;3:Patch-wise weight optimization of weight vectors ($\left\{W_{i}\right\}$) by multi-task sparse learning presented in Section 2.3.2;4:**Online Testing:**5:Active AU ($AU_{A}$) detection for testing samples by sparse representation presented in Section 2.3.3.

#### 2.3.1. AU Weighting

Motivated from the causal AU pair extraction with a large transition probability by the Bayesian network (BN) [46], the representative abilities of all of the AUs for each expression are weighted in this work to decrease the influence of weakly-related AUs and provide a constraint for the following patch-wise weight optimization.

With the labeled AUs of all of the training samples, the causal relation network between the AUs is obtained with the conditional probability matrix. The probability $p_{i|j}^{e}$ of the *i*-th AU conditioned on the *j*-th AU w.r.t. the *e*-th expression is defined as the product of the co-occurrence and co-absence probabilities as follows

(5) $$p_{i|j}^{e}=op_{i|j}^{e}\cdot ap_{i|j}^{e},$$

where the co-occurrence probability of the i,j-th AUs conditioned on the *j*-th AU is defined as the conditional probability:

(6) $$op_{i|j}^{e}=p\left(i\in AU^{e}|j\in AU^{e}\right),$$

where $AU^{e}$ denotes all of the action units of the *e*-th expression. Additionally, the co-absence probability of action units i,j conditioned on $j\notin AU^{e}$ is defined as the probability:

(7) $$ap_{i|j}^{e}=p\left(i\notin AU^{e}|j\notin AU^{e}\right),$$

Then, the degree of the causal relation of AU pair (i,j) is defined as follows:

(8) $$p_{i,j}^{e}=min\left(p_{i|j}^{e},p_{j|i}^{e}\right).$$

The ‘min’ function of two conditional probabilities is adopted to avoid abnormal probability resulting from the imbalance of expressions related with each AU. For example (see Figure 3), $AU_{2}$ is related to a large number of expressions, which may result in significantly larger arrival transition probability.

For each expression, a relation probability matrix of dimensions of 20×20 is obtained with Equation (8). With the causal relation matrices for all of the expressions, the representative ability of the AU for each expression can be found by simultaneously maximizing the representative ability for the considered expression and minimizing the representative abilities for the other expressions. That is, the representative ability of the AU pair (i,j) for the *e*-th expression is obtained as follows:

(9) $$RepAb_{i,j}^{e}=\left\{\begin{array}{cc} 1+\frac{1}{\left|\{l\neq e\}\right|}\left(p_{i,j}^{e}-\sum_{l\neq e}p_{i,j}^{l}\right) & \mathrm{if}\;\left\{i,j\right\}\subset AU^{e}\\ 0 & \mathrm{otherwise}. \end{array}\right.$$

where |{l≠e}| represents the number of elements in the set {l≠e}. Finally, the representative ability of the *i*-th AU for the *e*-th expression is obtained as follows:

(10) $$RA_{i}^{e}=\left\{\begin{array}{cc} \frac{1}{\left|\{j\neq i\}\right|}\sum_{j\neq i}RepAb_{i,j}^{e} & \mathrm{if}\;i\in AU^{e}\\ 0 & \mathrm{otherwise}. \end{array}\right.$$

For applying $\left\{RA_{i}^{e},1\leq i\leq 20,1\leq e\leq 7\right\}$ for recognition, the maximum AU representative ability of each expression and face part is collected, which are denoted as $RAP_{i}^{e}$ and presented as follows:

(11) $$RAP_{i}^{e}=\max_{j\in IA_{i}}RA_{j}^{e}$$

where the set $IA_{i}$ denotes the AU indices corresponding to the *i*-th part. As the correspondence between AU and face part (PT), the maximum representative abilities of the parts are used to weigh the corresponding AUs, which are denoted as $W_{AU}=\left\{RAP_{i}^{e},1\leq i\leq 7,1\leq e\leq 7\right\}$.

Due to the number limitation of AUs and the training samples, the representation space of AUs is limited when they are simply organized. In order to expand the representation space of the AUs, the contribution of the patches contained in each AU is weighted by the following weight optimization model in this work.

#### 2.3.2. Patch Weight Optimization

Based on the weights of AUs for each expression, a weight optimization model is proposed to weigh the patches of each AU for the considered expression triplet $G=\{e_{1},e_{2},e_{3}\}$.

The objective is to minimize a loss function with the weight sparseness and regularization constraints, which is presented as follows:

(12) $$\left\{\begin{array}{c} \begin{array}{c} \min_{w}E+{\lambda ||w||}_{1}=\frac{1}{N}\sum_{j}\sum_{k\in ID_{j}}\log\left(\frac{1}{1+exp(-\alpha \cdot g\left(f_{j},f_{k},w\right))}\right)+{\lambda ||w||}_{1},\\ s.t.||w_{PT_{i}}{\left|\right|}_{1}=RAP_{i}^{G}{\left||w|\right|}_{1},i={1,\cdots ,7;||w||}_{2}=1. \end{array} \end{array}\right.$$

where $f_{j},\;f_{k}$ are the features of the *j*-th and *k*-th training samples of the triplet *G*, whose patch feature is defined in Equation (2) and reduced to two dimensions using PCA and LDA [30]. $N=\sum_{j}\left|ID_{j}\right|$, and $ID_{j}$ records all of the training sample indices. Vector *w* records the weights of all of the patches; $w_{PT_{i}}$ records the weights of the patches related with the *i*-th part $PT_{i}$; parameter *α* is fixed to one. $RAP_{i}^{G}$ denotes the normalized representative abilities w.r.t. the considered expression triplet *G*, which is formulated as follows:

(13) $$RAP_{i}^{G}=\max_{1\leq j\leq 3}RAP_{i}^{e_{j}},RAP_{i}^{G}\leftarrow \frac{RAP_{i}^{G}}{\sum_{i}RAP_{i}^{G}}.$$

The loss function $g(f_{j},f_{k},w)$ reflects the similarity loss of the feature vectors $f_{j},\;f_{k}$ with the weight vector *w*, which is constructed to minimize the intra-class variance and maximize the inter-class variance as follows:

(14) $$g\left(f_{j},f_{k},w\right)=\left\{\begin{array}{c} \begin{array}{cc} <f_{j},f_{k}\cdot w>-<f_{j},f_{j_{0}}\cdot w> & \mathrm{if}\;L\left(f_{k}\right)\neq L\left(f_{j}\right)\\ <f_{j},f_{j_{0}}\cdot w>-<f_{j},f_{k}\cdot w> & \mathrm{if}\;L\left(f_{k}\right)=L\left(f_{j}\right)\\ \mathrm{with}\;j_{0}=arg\max_{\left\{t:L\left(f_{t}\right)=L\left(f_{j}\right)\right\}}<f_{j},f_{t}\cdot w> & \end{array} \end{array}\right.$$

where $L(f_{k})$ is the expression label of the training feature vector $f_{k}$.

For solving the optimization problem (12), the gradient of the first term of the minimization objective function in Equation (12) is formulated as follows:

(15) $$\frac{\partial E}{\partial w}=\frac{\alpha }{N}\Sigma_{j}\Sigma_{k\in ID_{j}}\frac{e_{j,k}\left(w\right)-1}{e_{j,k}\left(w\right)}\cdot \frac{\partial g(f_{j},f_{k},w)}{\partial w},$$

where $e_{j,k}\left(w\right)=1+exp\left(-\alpha \cdot g\left(f_{j},f_{k},w\right)\right)$ and $\frac{\partial g(f_{j},f_{k},w)}{\partial w}$ is computed based on Equation (14) as follows:

(16) $$\frac{\partial g(f_{j},f_{k},w)}{\partial w}=\left\{\begin{array}{c} \begin{array}{cc} f_{j}\cdot f_{k}-f_{j}\cdot f_{j_{0}} & \mathrm{if}\;L\left(f_{k}\right)\neq L\left(f_{j}\right)\\ f_{j}\cdot f_{j_{0}}-f_{j}\cdot f_{k} & \mathrm{if}\;L\left(f_{k}\right)=L\left(f_{j}\right). \end{array} \end{array}\right.$$

The sparseness term of Model (12) adopts the $L_{1}$ norm, which is a special case of the $L_{1}/L_{2}$ mixed-norm employed in the work [45,55]. Thus, the optimization model (12) is solved with the modified multi-task sparse learning algorithm employed in [45,55] with several differences presented as follows. The overall optimization algorithm is elaborated in Algorithm 2.

- The weight of each patch is initialized as a ratio of the corresponding AU representative ability as follows:
  (17) $$w_{P_{j},0}\leftarrow \frac{RAP_{i}^{G}}{|PT_{i}|},\;w_{0}\leftarrow \frac{w_{0}}{\left|\right|w_{0}{\left|\right|}_{2}},$$
  where *i* is the index of the part including the *j*-th patch, $|PT_{i}|$ denotes the number of patches in the part $PT_{i}$ and $w_{P_{j},0}$ records the weights of the *j*-th patch $P_{j}$. The initialization procedure is presented in Step 3 of Algorithm 2;
- The weight vector $w_{s+1}$ and auxiliary vector $v_{s+1}$ in the s+1-th iteration of Algorithm 2 are normalized to satisfy the constraint defined in Equation (12) as follows:
  (18) $$w_{P_{j},s+1}\leftarrow \frac{w_{P_{j},s+1}RAP_{i}^{G}}{\left|\right|w_{PT_{i},s+1}{\left|\right|}_{1}},w_{s+1}\leftarrow \frac{w_{s+1}}{\left|\right|w_{s+1}{\left|\right|}_{2}},$$
  where *i* is the index of the part including the *j*-th patch and $w_{PT_{i}}$ denotes the weights of the part $PT_{i}$. The normalization is employed in Steps 11 and 19;
- Compared with [45], optimization Model (12) is proposed by minimizing the feature similarity bias of different expression classes in Equation (14), which uses the information of mutual feature difference and contains more information than that of expression label matching in [45]. The corresponding objective function and the gradient vector are changed according to Equations (12) and (15), as revealed in Step 5.

With the weight optimization model (12) and Algorithm 2, the weights of the patches for each expression triplet are obtained. The number of optimized weight vectors $\left\{W_{i}\right\}$ equals $C_{7}^{3}=35$, i.e., the number of expression triplets.

**Algorithm 2** The modified multi-task sparse optimization.1:Obtain the feature vectors of the training and testing samples.2:Initialization: the coefficients $\lambda =\eta =1e^{-2},\;S=3e^{1},\;\epsilon =1e^{-10},\;MaxNumF=4$.3:Initialize the weight vector $w_{0}$ as in Equation (17).4:**for** s=0,⋯,S **do**5: Compute the objective value FunV with Equation (12) and the gradient $\frac{\partial E}{\partial w_{s}}$ with Equation (15).6: **if** s≥2 AND FunV−pFunV≥−ϵ **then**7:  NumF←NumF+1.8: **else**9:  NumF←0.10: **end** **if**11: Perform $w_{s+1}=v_{s}-\eta \frac{\partial E}{\partial w_{s}}$, normalize current weight vector $w_{s+1}$ with Equation (18). Renew the *i*-th element of the weight vector $w_{s+1}$ as follows.12: **if** $|w_{i,s+1}|\geq \lambda \eta$ **then**13:  $w_{i,s+1}=\left(1-\frac{\lambda \eta }{|w_{i,s+1}|}\right)w_{i,s+1}$14: **else**15:  $w_{i,s+1}=0$16: **end** **if**17: $a_{s+1}=\frac{2}{s+3}$, $\delta_{s+1}=w_{s+1}-w_{s}$, pFunV←FunV18: $v_{s+1}=w_{s+1}+\frac{1-a_{s}}{a_{s}}a_{s+1}\delta_{s+1}$19: Normalize weight vectors $w_{s+1},\;v_{s+1}$ with Equation (18).20: **if** $\left|\right|\delta_{s+1}{\left|\right|}_{2}\leq \epsilon$ OR NumF>MaxNumF **then**21:  break22: **end** **if**23:**end for**

#### 2.3.3. Active AU Detection

**Figure 4:**
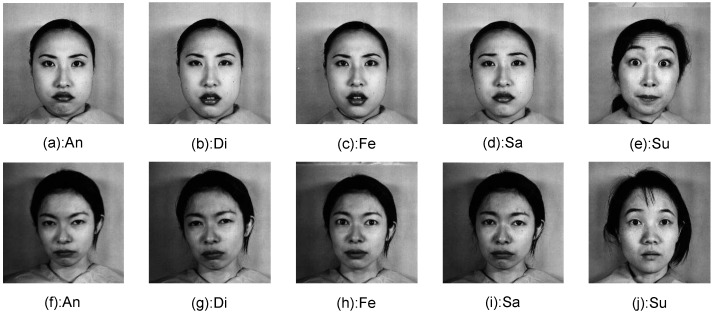
Expression examples with similar appearance from the Jaffe database.

Though each expression is related with several AUs, these AUs may not be present at the same time. For example, while the AUs involving brows and eyes are present for the surprise expression shown in Figure 4e,j, the AU involving mouth was less active. In this case, error may occur if the features extracted from the AU involving mouth are included for expression recognition. To address this issue, we proposed a sparse representation-based approach to identify the parts where the corresponding AUs are active for each testing sample before expression recognition.

For the *k*-th part of the *i*-th testing sample in Figure 3, the sparse representation is represented as follows:

(19) $$\min_{c^{\left(k\right)}}\frac{1}{2}\left|\right|f_{i}^{\left(k\right)}-D^{\left(k\right)}c^{\left(k\right)}{\left|\right|}_{2}^{2}+\lambda ||c^{\left(k\right)}{\left|\right|}_{1}.$$

where $f_{i}^{\left(k\right)}$ is the vectorized features of the *i*-th testing sample and $D^{\left(k\right)}=\left[f_{tr_{1}}^{\left(k\right)},f_{tr_{2}}^{\left(k\right)},\cdots ,f_{tr_{n}}^{\left(k\right)}\right]$ are the patch features corresponding to the *k*-th face part ($PT_{k}$) of all of the training samples of the candidate expression triplet and the neutral expression. Weight vector $c^{\left(k\right)}$ records the *n*-dimensional sparse representation coefficients, and *λ* is the regularization parameter set as $1e^{-3}$ in this work [56,57].

With the part-based sparse representation, the coefficients w.r.t. the AUs related with neutral and non-neutral expressions are obtained, where the AUs related with neutral expression ($AU_{NE}$) are $\left\{AU_{1},AU_{4},AU_{8},AU_{13},AU_{15},AU_{17},AU_{19}\right\}$ and the others are the AUs related with non-neutral expressions ($AU_{EX}$) as presented in Figure 3. More precisely, for the *k*-th part of the weight vector $c^{\left(k\right)}$, the corresponding weight components w.r.t. the AUs related with neutral and non-neutral expressions are obtained as follows:

(20) $$\left(c_{NE,j}^{\left(k\right)},c_{EX,j}^{\left(k\right)}\right)=c_{j}^{\left(k\right)}\cdot \left(r_{NE,j}^{\left(k\right)},r_{EX,j}^{\left(k\right)}\right).$$

where $r_{NE,j}^{\left(k\right)},\;r_{EX,j}^{\left(k\right)}$ are the number ratios of the *j*-th feature element of the training samples w.r.t. the AUs of neutral and non-neutral expressions, respectively. That is, the weight vector $c^{\left(k\right)}$ is grouped into the sub-vectors $c_{NE}^{\left(k\right)},\;c_{EX}^{\left(k\right)}$ with neutral and non-neutral expressions. Finally, the activeness of $PT_{k}$ of the *i*-th testing sample is defined as follows:

(21) $$ActV_{i}^{\left(k\right)}=\frac{1}{n}\sum_{j=1}^{n}c_{EX,t_{j}}^{\left(k\right)}-c_{NE,t_{j}}^{\left(k\right)}$$

where $t_{j}$ is the index of the weight with the the *j*-th largest value in the vector $c_{EX}^{\left(k\right)}$ or $c_{NE}^{\left(k\right)}$ and n=10 is the number of patches set to reduce the influence of abnormal weight components by sparse representation (19).

To judge whether $PT_{k}$ or the corresponding AU is active or not, we treat each training sample $f_{i}$ with a non-neutral label as the testing sample and obtain its activeness value $TrActV_{i}^{\left(k\right)}$ of $PT_{k}$ with Equation (21), where the part feature of $f_{i}^{\left(k\right)}$ is removed from the dictionary $D^{\left(k\right)}$ when obtaining the sparse coefficients $c^{\left(k\right)}$ in Equation (19). Finally, the considered part of the testing image is decided to be active if $ActV_{i}^{\left(k\right)}$ is larger than the average of training activeness values as follows:

(22) $$fg_{i}^{\left(k\right)}=\left\{\begin{array}{c} \begin{array}{cc} 1 & \mathrm{if}\;ActV_{i}^{\left(k\right)}\geq \frac{1}{n_{tr}}\sum_{j=1}^{n_{tr}}TrActV_{j}^{\left(k\right)}\\ 0 & otherwise. \end{array} \end{array}\right.$$

where $n_{tr}$ is the number of training samples with non-neutral labels. When the number of the selected active AUs for the *i*-th testing sample is less than two, which is likely to happen for the neutral expression sample, then the AUs with the top two largest activeness values $ActV_{i}^{\left(k\right)}$ presented in Equation (21) are determined to be active.

**Figure 5:**
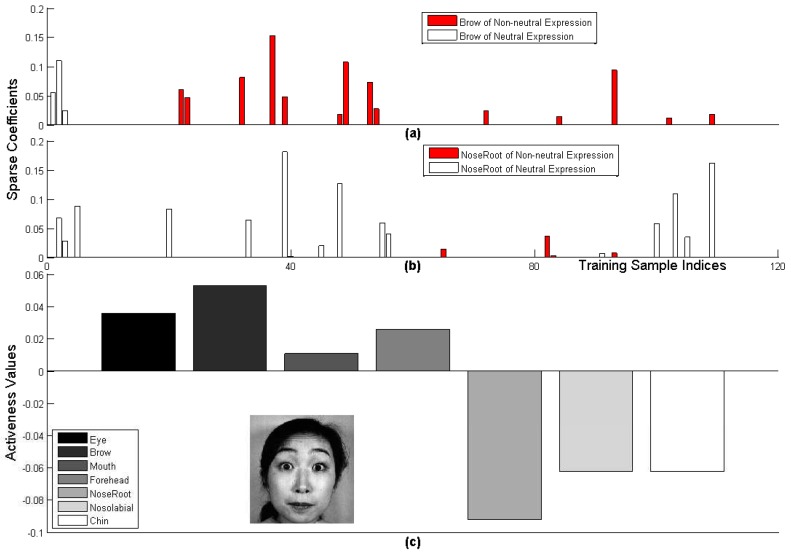
The sparse coefficients and the activeness values of an example surprise expression. (**a**,**b**) The sparse representation coefficients of ‘Brow’ and ‘NoseRoot’; (**c**) the example surprise expression and the activeness values of its seven parts.

Figure 5 presents the sparse representation coefficients and the activeness values for an example surprise expression, where Figure 5a,b present the sparse coefficients corresponding to the active ‘Brow’ and the non-active ‘NoseRoot’. The training dictionary of the testing sample consists of 27 neutral expressions and 82 non-neutral expressions such as surprise, laugh and fear. Figure 5a,b show that the number of non-zero coefficients corresponding to non-neutral expression samples for active ’Brow’ is significantly larger than that for non-active ’NoseRoot’. Figure 5c shows the activeness values of the seven parts of the same example, which clearly suggests that ‘Brow’ is the most active part and ‘NoseRoot’ is the most non-active part. While ‘Brow’, ‘Eye’ and ‘Forehead’ are decided to be active and included for feature representation, non-active parts, like ‘Mouth’, ‘NoseRoot’, ‘Nasolabial’ and ‘Chin’ regions, will not be involved in the following expression recognition.

After the active AU detection for each testing sample, the optimized patch weights for the corresponding candidate expression triplet with Algorithm 2 are used to weigh the selected active AUs (AU${}_{A}$) and the involved patches for the following recognition.

### 2.4. Weighted SVM for Classification

After the feature weight optimization and AU activeness detection, support vector machine (SVM) with a slightly modified kernel function is employed for the classification [58]. Rather than treat the feature weights as variables in SVM and obtaining them with mutual information [59], the optimized feature weights learned in Section 2.3.2 are directly integrated with the patches involved with the detected active AUs in Section 2.3.3 for the recognition. That is, the new inner product ${\langle f_{i},\;f_{j}\rangle }_{w}$ of two features $f_{i},\;f_{j}$ with weight vector *w* is defined as follows:

(23) $${\langle f_{i},\;f_{j}\rangle }_{w}=\langle f_{i},\;w\cdot f_{j}\rangle .$$

where $\langle x,\;y\rangle =x^{T}y$ is the inner product of two vectors and $x\cdot y=(x_{1}y_{1},\cdots ,x_{n}y_{n})$ is the dot product of two vectors. Finally, with the new defined inner product (23) as the kernel function, SVM is used for the recognition.

## 3. Experimental Results

We perform the experiments using MATLAB 2014b on a PC with a 4-GHZ core processor and 32 GB RAM. For the experimental testing, the Jaffe [60], Cohn–Kanade (CK+) [61] and SFEW2 [62] databases are employed for the performance and feature optimization study. Another three databases, i.e., Taiwanese Facial Expression Image Database (TFEID) [63], Yale-A database (YALE) [64] and EURECOM [65], are employed for the generalization testing. Among them, the database SFEW2 was collected in the real life, and the faces were captured with un-controlled head poses and lighting conditions. Actually, the appearance of the same expression is different from person to person, to guarantee that the image really represents a specific expression, these collected expressions are labeled by two independent labellers [62]. The remaining databases were videoed in the controlled lighting condition, and the faces are all frontal; the corresponding participants were instructed by an experimenter to perform a series of facial displays for each expression [61].

The Jaffe database consists of 213 expression images of 10 Japanese female models, which can be categorized into six basic and the neutral expressions, i.e., angry (An), disgust (Di), fear (Fe), happy (Ha), sad (Sa) and surprise (Su). The CK+ database consists of 593 expression sequences from 123 subjects, where 327 sequences are labeled with one of seven expressions (angry, disgust, fear, happy, sad, surprise and contempt). Each sequence contains a set of captured frames when the subject changes his/her expression; 1033 expression images, i.e., the neutral and three non-neutral images sampled from each expression sequence are used for testing. The database SFEW2 is derived from the sub-challenge of static expression recognition in The Third Emotion Recognition in the Wild Challenge [62], which includes 958, 436 and 372 training, validation and testing samples of seven basic expressions. As the labels of the testing set are not publicly available, the validation set was used in this paper for testing. The images were videoed in the un-controlled condition with different lighting, head poses, profiles, resolutions and face colors. Five landmark points were located with [50,66] for face alignment.

**Figure 6:**
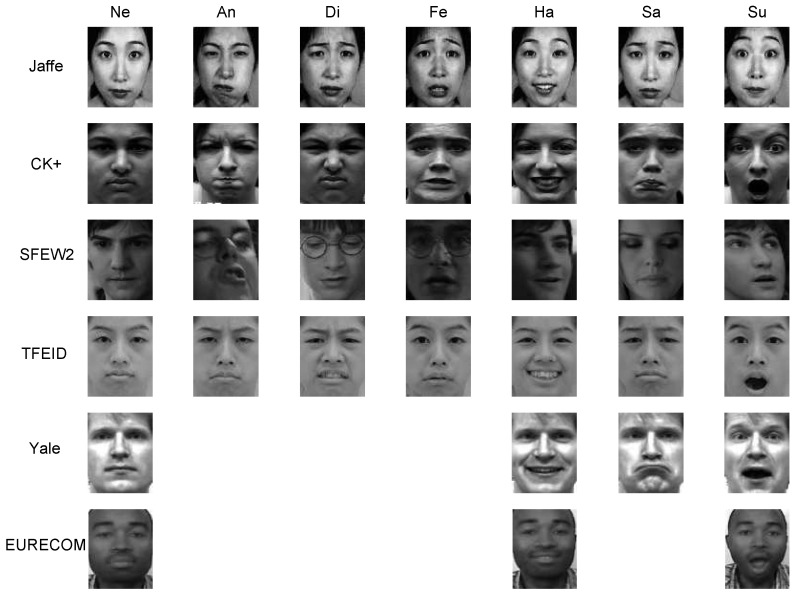
Examples of six expression databases. Neutral (NE), angry (An), disgust (Di), fear (Fe), happy (Ha), sad (Sa) and surprise (Su).

The Taiwanese Facial Expression Image Database (TFEID) database consists of 268 expression images from 40 subjects (20 females, 20 males); each of the subject presents six basic expressions and the neutral expression. The Yale expression database consists of 60 expression images from 15 subjects; each of the subject presents three basic expressions (happy, sad and surprise) and the neutral expression. The EURECOM Kinect Face Dataset (EURECOM) consists of 312 expression images from 52 subjects (14 females, 38 males); each subject presents two basic expressions (happy and surprise) and the neutral expression. The expression images are captured in two sessions at an interval of about two weeks. The six basic and the neutral examples of the six databases are demonstrated in Figure 6. For the following experiment, the person-independent strategy with ten-fold setting is employed for testing and comparison. More precisely, the considered database is divided into ten groups with approximately an equal number of person IDs. While nine of them were used for training, the remaining group was used for testing. The process was randomly repeated ten times, and the average accuracy is recorded as the final result.

### 3.1. Number of Candidate Expressions Suggested by the First Stage Classifier

Take the Jaffe database as an example, some expressions in the dataset are quite difficult to discriminate, even for human eyes. For example, the angry and sad expressions shown in Figure 4a,d,f,i are very similar. It would be more plausible to develop a hierarchy system, which could discriminate the easy categories at the first stage, and then differentiate the difficult categories at the second stage.

**Figure 7:**
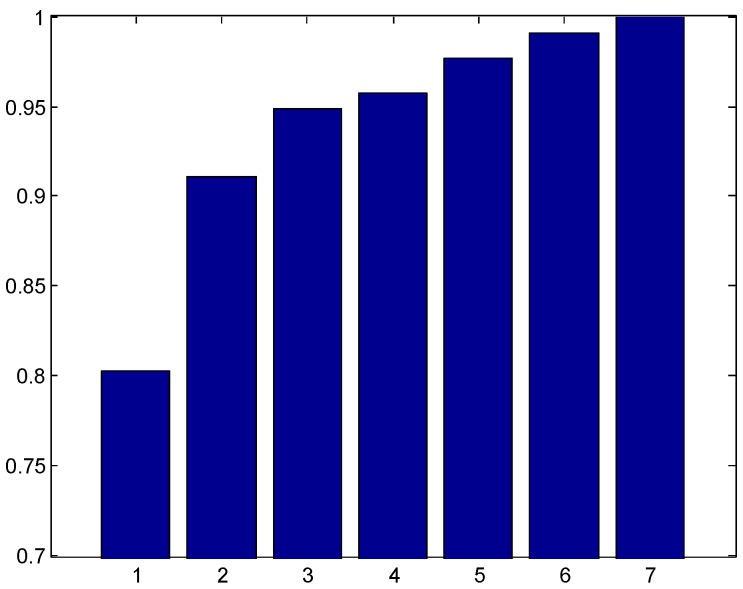
The variation of recognition rates with the value of ranks for the Jaffe database.

To decide the number of candidate expressions proposed by the first stage classifier, we show in Figure 7 the variation of the accuracy with the value of *k* when the top-*k* strategy is adopted for expression recognition. A classification is said to be correct if one of the top-*k* labels returned by the system matches the true label of the sample. The accuracy generally increases with the values of rank, *k*. While the accuracy of 91.5% was achieved for k=2, the accuracy reached 96% for k=3. To reach a trade-off between accuracy and efficiency, we set k=3 for the first stage classification, i.e., the top three expression labels were assigned for the testing sample at the first stage. Based on the three candidates, the final label was given by a different model trained using finer features at the second stage.

### 3.2. Recognition Performance Analysis

To evaluate the effects of different models like AU weights, patch weight optimization and active AU detection, we tested the performance of the recognition system with/without those models on the Jaffe, CK+ and SFEW2 databases. For traditional one-stage recognition, the features extracted from each patch were concatenated (see Equation (4)) and input to SVM for classification. The feature was further optimized using the proposed AU weight, patch weight optimization and active AU detection. The recognition performance of the system for different models is tabulated in Table 2. One can observe from the table that the proposed models significantly boost the performance. For example, when all three models were used, the recognition performance increased from 82.63% to 89.67%, from 89.06% to 94.09% and from 42.2% to 46.1%, for the Jaffe, CK+ and SFEW2 datasets, respectively.

**Figure 8:**
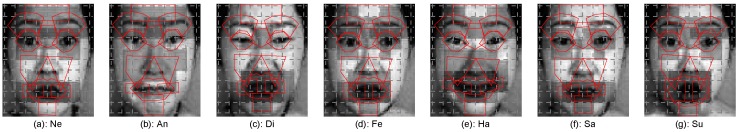
The top three most representative parts for each expression. The darker regions denote the larger representative abilities.

Figure 8 shows the top three most representative AUs of each expression; one can observe from the figure that the most representative parts of the surprise expression (g) are the brows, eyes and mouth. The most representative regions of the laugh expression (e) are the brow, mouth and nasolabial parts.

**Figure 9:**
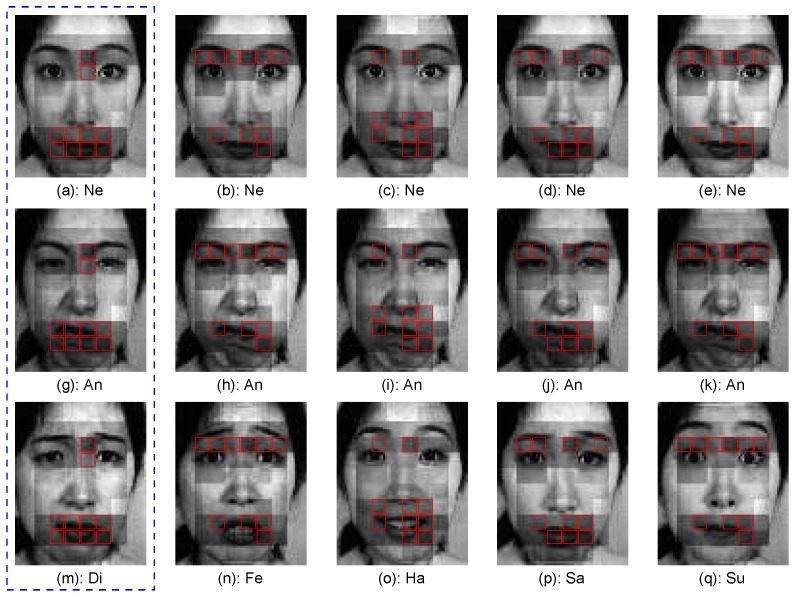
The visualization of optimized patch weights for five expression triplets. The darker the patch is, the larger is the weight. The patches with the top ten largest weights are labeled with red rectangles.

To analyze the performance of the patch weight optimization, Figure 9 depicts the optimized weight vectors of five expression triplets of the Jaffe database. It can be seen from the figure that the weights of the patches of each AU are further optimized. With the proposed weight optimization, the discrimination ability of the weighted patches for expressions with small variation is increased, and performance improvements on the databases Jaffe and CK+ are observed in the fifth column of Table 2.

**Figure 10:**
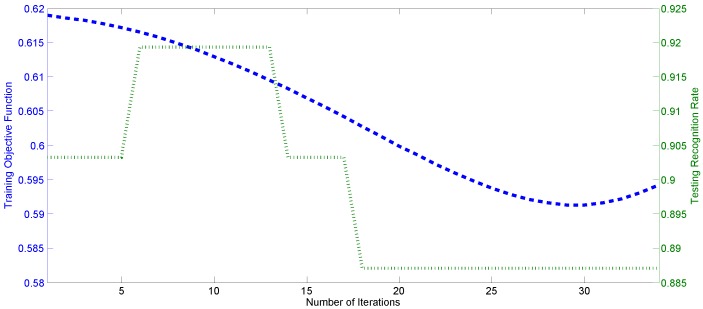
The evolutions of the objective function values and the testing recognition rates of expression triplet: fear, angry and sad w.r.t. the number of iterations.

Due to the limited number of training samples, the weight optimization is not always beneficial to the recognition rate improvement. Figure 10 demonstrates the variation of the objective function values in Equation (12) and the testing accuracy of an example expression triplet (angry, fear and sad) w.r.t. the number of iterations on the Jaffe database, when active AU detection was not applied. It can be seen that the recognition rate is not always increasing with the descendant of the objective function values due to the difference between the testing and training samples. Thus, active AUs could be detected to represent the specific features for each testing expression sample.

**Figure 11:**
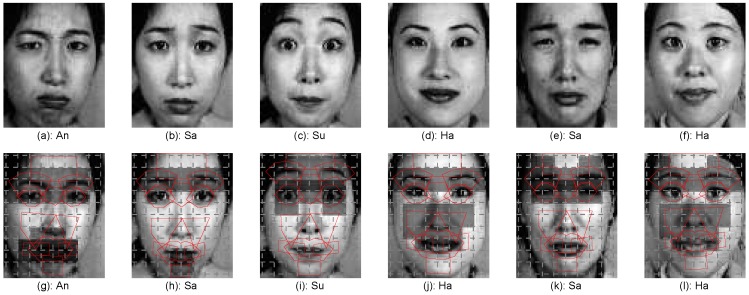
The top two active AUs of six example testing images.

To study the effect of the active AU detection for recognition, Figure 11 presents the top two active AUs of six example testing expressions, where Figure 11c,i show that the brow and eye parts are more active than the other parts for the expression sample presented in Figure 4e. When these active parts are used for the feature encoding, the expression samples will be correctly recognized.

To analyze the algorithm performance overall, the confusion matrix of the final recognition results on the databases Jaffe and CK+ is presented in Table 3 and Table 4, respectively. Both tables show that the angry, fear and sad expressions are relatively difficult to be correctly recognized. The difficulty is verified by the expressions presented in Figure 4, where faces present similar features, not only in the appearance, but also in the face part deformation. Table 4 suggests that the sad expression is mostly misclassified as the neutral expression (error rate 14.28%).

### 3.3. Feature Optimization Comparison

This section mainly compares the performance of the proposed weight optimization algorithm with other related algorithms, such as AdaBoost [19,20,26,35], linear discriminant analysis (LDA) [30,43], the chi square statistic (CSS) [48], multi-task salient patch selection (MTSPS) [45] and the uniform weights (UWs) setting. For the AdaBoost feature selection [35], the strong classifier of the final recognition is linearly composed of a number of patch-based weak classifiers. In the expression recognition [48], only the chi square statistic for weight assignment is employed. In the feature selection [43], the patch saliency score is related with the classification accuracy of the training expression samples, where PCA and LDA are employed to reduce the feature dimension. The salient feature selection in [45] trains a set of active common and specific expression patches. The same strategy of the triplet mode and the GSF feature is employed for a fair comparison. The recognition rates obtained by these algorithms on the databases Jaffe and CK+ are presented in Table 5.

Table 5 shows that the recognition rates of AdaBoost and LDA are lower than that of UWs. CSS achieves slightly better performance than UWs on the Jaffe database. In these models, the specificity of each expression and the causal relation information among AUs are not sufficiently exploited. To reduce the effects of personal ID information, the salient feature selection in [45] integrated the common and specific expression features, and higher recognition rates are achieved.

Different from the other feature selection algorithms, the AU-based feature optimization in the proposed algorithm weighs the AUs and the corresponding patches with the conditional transient probability matrix. The discrimination information contained in both large-scale AUs and small-scale patches is considered. Moreover, active AUs of each testing expression sample are also detected for the feature encoding. The best recognition rates achieved in Table 5 justified the advantages of the proposed feature optimization.

### 3.4. Comparison with the State-Of-The-Art

In this section, a comparison of the overall recognition rates with a number of the state-of-the-art algorithms is conducted. To make the comparison fair, the competing algorithms were all tuned for the best performance. The comparison results on the databases Jaffe, CK+ and SFEW2 are demonstrated in Table 6, Table 7 and Table 8, respectively, where the algorithm description, the category, the number of subjects, testing protocol and the final recognition rates are considered.

For the Jaffe database, our proposed algorithm achieves a competitive recognition rate among all of the algorithms in Table 6. The algorithm [41] using the deep belief network yields the highest recognition rate of 91.8%. However, feature selection and classifier training are time consuming, and the process requires several days for each database. Rather than using a well-designed feature representation, the proposed algorithm achieves the best accuracy of 89.67% as the radial feature-based algorithm [22] among the traditional algorithms. For the CK+ database, the proposed algorithm achieves the highest accuracy of 94.09%. As we are focused on seven-class expression recognition, those works developed for six expressions, like [21,37,41,43,44,45,49], are not included for comparison in this paper.

The feature and classifier adopted in the proposed algorithm are significantly different from the convolutional neural network (CNN)-based algorithms. In the following, the database (SFEW2) collected in real life is taken to compare the overall performance between CNN and the proposed algorithms. As SFEW2 was used in Emotion Recognition in the Wild Challenge for performance evaluation, we directly take the accuracies of participants for comparison. All of the top three participants adopted CNN, and their results are listed in Table 8, together with that of our approach.

While our approach achieves the top performance for the CK+ database, CNN-based methods perform much better for the real life dataset, i.e., SFEW2. As CNN-based algorithms employ randomly-cropped face regions for dataset augmentation, they are less sensitive to the face misalignment than the traditional algorithms. However, when a large training dataset is not available and the images were mostly frontal faces, e.g., Jaffe and CK+, the traditional approaches could perform better than CNN-based approaches. Furthermore, the network and parameters of CNN need to be finely tuned, which is much more time consuming than traditional algorithms.

### 3.5. Cross-Database Performance Study

To study the generalization ability of the proposed model, cross-database experiments are conducted, and the corresponding recognition rates are presented in Table 9. In this testing, while one database is set as the training set, the other database is used as the testing set for evaluation.

It can be seen from Table 9 that the radial feature encoding [22] with the probability projection achieves the highest accuracy when the databases Jaffe and CK+ are used for testing and training, respectively. The proposed algorithm achieves a competitive recognition rate of 46.01%, which is better than the recognition rate of 32.86% achieved by [22] when the employed probability projection is replaced with the Borda count strategy. When Jaffe is used as the training and CK+ is used for testing, the proposed algorithm also achieves competitive accuracy.

To further study the generalization ability of the proposed model, the databases of CK+ and Jaffe are used as the training, while one of the other three databases is chosen for the testing. The accuracy is presented in the last three columns of Table 9, which shows that the proposed algorithm achieves a much better recognition rate than the algorithm [22] on the database TFEID and a competitive recognition rate on the database YALE.

## 4. Discussion and Conclusions

In this work, a two-stage expression recognition model based on triplet-wise feature optimization is proposed; the novelty of the this work is concentrated on three aspects. First, overall facial expression recognition is transformed into the triplet-wise mode to sufficiently exploit the specificity of each expression. Second, AU weighting and patch weight optimization are proposed for each expression triplet. Lastly, the online detection of active AUs is proposed for each testing expression sample to reduce the influence of the non-active features in recognition. Experimental results and a comparison with the related state-of-the-art algorithms verify the effectiveness and competitiveness of the proposed algorithm.

Although competitive results are obtained with the proposed model, this still leaves room for further improvement. First, feature optimization of more than two stages can be explored for the performance improvement. Second, more efficient features should be devised and integrated into the feature optimization model. Third, the cross-database recognition rates are still not high enough for the real application, which will be explored in our future work. Lastly, the ideas of AU weighting, feature sparseness optimization and active AU detection can be combined with CNN-based algorithms to improve the feature encoding based on face frontalization [71].

## Figures and Tables

**Table 1:** Feature point sequences for constructing the RFS regions in Figure 2c and the region sizes in Figure 2d. Take $R_{1}$ as an example; points $\left\{P_{3},P_{8}\right\}$ define the length and width $d_{1}$ of $R_{1}$.

$R_{1}$	$R_{2}$	$R_{3}$	$R_{4}$
$\left\{P_{3},P_{8}\right\}$, $d_{1}=\frac{1}{2}\left|\right|P_{3}-P_{8}{\left|\right|}_{2}$	$\left\{P_{1}-P_{5}\right\}$, $d_{2}=2||P_{1}-P_{2}{\left|\right|}_{2}$	$\left\{P_{6}-P_{10}\right\}$, The same as $d_{2}$	$\left\{P_{20}-P_{25}\right\}$, $d_{3}=2||P_{20}-P_{21}{\left|\right|}_{2}$
$R_{5}$	$R_{6}$	$R_{7}$	$R_{8}$
$\left\{P_{26}-P_{31}\right\}$, The same as $d_{3}$.	$\left\{P_{32}-P_{43}\right\}$, $d_{8}=4||P_{32}-P_{33}{\left|\right|}_{2}$	$\left\{P_{12},P_{23},P_{5},P_{6},P_{26}\right\}$	$\left\{P_{32},P_{33},P_{43}\right\}$, $d_{6}=2||P_{32}-P_{43}{\left|\right|}_{2}$, $d_{7}=\left|\right|P_{33}-P_{43}{\left|\right|}_{2}$
$R_{9}$	$R_{10}$	$R_{11}$	$R_{12}$
$\left\{P_{37},P_{38},P_{39}\right\}$, The same as $d_{6},d_{7}$	$\left\{P_{32},P_{13},P_{20},P_{15}\right\}$, $d_{4}=\left|P_{20}^{x}-P_{13}^{x}\right|$, $d_{5}=\left|P_{13}^{y}-P_{15}^{y}\right|$	$\left\{P_{38},P_{13},P_{29},P_{15}\right\}$, The same as $d_{4},d_{5}$	$\left\{P_{39},P_{43}\right\}$, $d_{9}=\left|\right|P_{43}-P_{39}{\left|\right|}_{2}$

**Table 2:** The effects of different models on the overall recognition rate (%). CK+, Cohn–Kanade.

Database	Seven Expression	Triplet-Wise-Based Two-Stage Classification			
	Patch Feature	Patch Feature	Patch Feature+ AU Weight	Patch Feature+ AU Weight+ Patch Weight	Patch Feature+ AU Weight+ Patch Weight+ Active AU Detection
Jaffe	82.63	83.10	84.04	86.85	**89.67**
CK+	89.06	89.55	91.09	93.32	**94.09**
SFEW2	42.2	42.2	42.66	43.81	**46.1**

**Table 3:** Confusion matrix (%) of the proposed recognition algorithm on the Jaffe database.

Expression	Ne	An	Di	Fe	Ha	Sa	Su
Ne	**90**	3.33	0	0	0	0	6.67
An	0	**83.33**	6.67	0	0	10	0
Di	0	3.45	**93.1**	0	3.45	0	0
Fe	3.13	0	0	**87.5**	6.25	3.12	0
Ha	0	0	0	0	**100**	0	0
Sa	0	6.45	3.23	6.45	6.45	**77.42**	0
Su	0	0	0	0	3.33	0	**96.67**

**Table 4:** Confusion matrix (%) of the proposed recognition algorithm on the CK+ database.

Expression	Ne	An	Di	Fe	Ha	Sa	Su
Ne	**94.34**	2.84	0	0	0.94	0.94	0.94
An	9.63	**85.93**	4.44	0	0	0	0
Di	0	0	**100**	0	0	0	0
Fe	2.67	0	0	**86.67**	5.33	1.33	4
Ha	0	0	0	0	**100**	0	0
Sa	14.28	1.2	0	0	0	**84.52**	0
Su	2.81	0	0	0	0	0	**97.19**

**Table 5:** The recognition rates (%) of different feature selection algorithms on two databases. UWs, uniform weights; CSS, chi square statistic; MTSPS, multi-task salient patch selection.

Database	UWs (Uniform Weights)	AdaBoost [35]	LDA [43]	CSS [48]	MTSPS [45]	Ours
Jaffe	83.10	81.22	82.63	83.57	85.45	**89.67**
CK+	89.55	88.58	90.71	90.22	92.45	**94.09**

**Table 6:** Comparison of different algorithms on the Jaffe database.

Algorithm	Category	Subjects	Protocol	Recognition Rate (%)
Feature and Classifier Selection [42]	Traditional	10	10-fold	85.92
Radial Feature [22]	Traditional	10	10-fold	89.67
Supervised LLE [33]	Traditional	10	10-fold	86.75
Ours	Traditional	10	10-fold	89.67
Deep CNN [11]	Deep learning-based	10	10-fold	88.6
Deep Belief Network [41]	Deep learning-based	10	10-fold	**91.8**

**Table 7:** Comparison of different algorithms on the CK+ database.

Algorithm	Category	Subjects	Protocol	Recognition Rate (%)
Maximum Margin Projection [32]	Traditional	100	5-fold	89.2
Feature Selection with GMM [67]	Traditional	97	10-fold	89.1
SVM (RBF) and Boosted-LBP [20]	Traditional	96	10-fold	91.4
Radial Feature [22]	Traditional	94	10-fold	91.51
Ours	Traditional	106	10-fold	**94.09**
AU Deep Network [47]	Deep learning-based	118	10-fold	92.05
Deep Neural Network [68]	Deep learning-based	106	5-fold	93.2

**Table 8:** The accuracy (%) of different algorithms on the SFEW2 database.

Pyramid of Histogram of Gradients+Local Phase Quantization + Non-linear SVM [62]	Hierarchical Committee CNN [12]	Multiple CNN [69]	Transfer Learning Based CNN [70]	Ours
35.93	**56.4**	56.19	48.5	46.1

**Table 9:** Comparison of the cross-database recognition rates (%). TFEID, Taiwanese Facial Expression Image Database; LPP, locality preserving projection.

Algorithm	CK+ Training	Jaffe Training	CK+ and Jaffe Training		
	Jaffe Testing	CK+ Testing	TFEID	YALE	EURECOM
SVM and LBP [20]	41.3	-	-	-	-
Radial Feature [22]	**55.87**	**54.05**	61.94	60.66	-
LPP [40]	30.52	27.97	-	-	-
SR [36]	40.5	-	-	-	-
Ours	46.01	47.05	**78.73**	**63.33**	43.27
